# Can Milrinone Be a Therapeutic Alternative in Persistent Pulmonary Hypertension of the Newborn? A Case Series and Narrative Review

**DOI:** 10.3390/pediatric17060116

**Published:** 2025-11-03

**Authors:** Eliza Wasilewska, Norbert Dera, Łukasz Minarowski, Łukasz Osiński, Anna Doboszynska, Sławomir Szajda, Alina Minarowska

**Affiliations:** 1 Department of Allergology, Medical University of Gdańsk, 80-211 Gdańsk, Poland; 2 Department of Obstetrics, Perinatology and Neonatology, Center of Postgraduate Medical Education, 01-809 Warsaw, Poland; 3 Warsaw Institute of Women’s Health, 00-189 Warsaw, Poland; 4 Head of Department of Respiratory Pathophysiology, Faculty of Health Sciences, Medical University of Bialystok, 15-540 Białystok, Poland; 5 2nd Department of Lung Diseases, Lung Cancer and Internal Diseases, University Teaching Hospital, 15-540 Bialystok, Poland; 6 Department of Neonatology and Neonatal Intensive Care, Regional Specialist Hospital in Olsztyn, 10-452 Olsztyn, Poland; 7 Department of Pulmonology, School of Public Health, Collegium Medicum, University of Warmia and Mazury in Olsztyn, 10-719 Olsztyn, Poland; 8 Department of Emergency Medicine, University of Warmia and Mazury, 10-719 Olszty, Poland; 9 Outpatient Cystic Fibrosis Clinic, Department of Pediatrics, Gastroenterology, Hepatology, Nutrition, Allergology and Pulmonology, Children’s University Hospital in Bialystok, 15-274 Bialystok, Poland

**Keywords:** persistent pulmonary hypertension of the newborn, PPHN, milrinone, inhaled nitric oxide, neonatal pulmonary hypertension, vasodilator therapy, phosphodiesterase inhibitors, case series, literature review, preterm infant

## Abstract

Background: Persistent pulmonary hypertension of the newborn (PPHN) remains a life-threatening condition resulting from failure of postnatal circulatory adaptation. Inhaled nitric oxide (iNO) is the standard first-line therapy; however, limited access or inadequate response highlight the need for alternative treatments. Milrinone, a selective phosphodiesterase-3 inhibitor with nitric oxide-independent vasodilatory and inotropic properties, has been proposed as one such option. Methods: In this study we present a case series of three neonates with PPHN—term (41 weeks), late preterm (35 weeks), and extremely preterm (23 weeks)—treated with intravenous milrinone in a neonatal unit without immediate access to iNO. A narrative literature review was also conducted, focusing on clinical outcomes, safety, and therapeutic applicability. Results: Milrinone was initiated within the first 24 h of life. In the term and late-preterm infants, oxygenation and echocardiographic parameters improved within 48 h, with normalization of shunt direction and successful extubation by days 4–10. Transient systemic hypotension occurred in both cases and required dose adjustment or vasoactive support. In the extremely preterm neonate, only temporary hemodynamic improvement was achieved, followed by severe intraventricular hemorrhage and coagulopathy, possibly exacerbated by vasodilatory and antiplatelet effects of milrinone. Conclusions: Milrinone may serve as a feasible adjunct or bridging therapy for PPHN when iNO is unavailable. However, its use requires careful hemodynamic and neurological monitoring, particularly in very preterm infants. Further studies are needed to confirm safety and define optimal dosing across gestational ages.

## 1. Introduction

Persistent pulmonary hypertension of the newborn (PPHN) remains a severe and life-threatening condition in neonatal medicine. It results from the failure of pulmonary circulation to adapt after birth, leading to persistently elevated pulmonary vascular resistance. This condition causes right-to-left shunting through the ductus arteriosus or foramen ovale, resulting in profound hypoxemia despite adequate lung ventilation [1,2]. The incidence of PPHN is estimated at 0.4–6.8 per 1000 live births, while in extremely preterm infants—particularly those with respiratory distress syndrome (RDS), sepsis, or intrauterine growth restriction—it may reach up to 8.1 per 1000 live births [1,2,3].

Well-documented risk factors include meconium aspiration syndrome (MAS), cesarean delivery, perinatal asphyxia, perinatal infections, pulmonary hypoplasia, male sex, low birth weight, and maternal conditions such as diabetes, obesity, or pregnancy-induced hypertension [4,5].

In addition to general supportive management (optimizing intravascular volume, nutrition, electrolyte and acid-base balance, thermoregulation, and adequate ventilation and oxygenation), inhaled nitric oxide (iNO) remains the first-line therapy for PPHN due to its proven efficacy in improving oxygenation. Early randomized controlled trials demonstrated improved oxygenation (PaO_2_ ≥ 20 mmHg) in approximately 60% of neonates [6], while more recent data suggest a response rate of up to 60–70% [7,8].

The use of iNO significantly reduces the combined risk of extracorporeal membrane oxygenation (ECMO) or death by approximately 34% (RR 0.66; 95% CI 0.57–0.77) [9]. However, analyses evaluating iNO’s impact on mortality alone have not shown a significant difference compared to controls (OR 1.04; 95% CI 0.59–1.82) [10,11,12]. This suggests that while iNO improves oxygenation and reduces the need for ECMO, it does not directly reduce mortality in this population. Despite current treatment strategies, a subset of patients remains unresponsive to therapy. Moreover, access to iNO may be limited, especially in smaller centers or in developing countries. In refractory cases, ECMO is considered, although it carries significant risks, costs, and technical demands [5].

Among pharmacologic alternatives, attention has turned to milrinone, a selective phosphodiesterase type 3 (PDE3) inhibitor [13]. Milrinone increases intracellular cyclic adenosine monophosphate (cAMP) in cardiomyocytes and vascular smooth muscle cells, providing both inotropic and vasodilatory effects, including pulmonary vasodilation. International guidelines, including those from the American Heart Association (AHA) and American Thoracic Society (ATS), suggest considering milrinone in cases of cardiac dysfunction or lack of response to iNO [1,2]. However, they emphasize that available evidence remains limited, and milrinone should be regarded as a second-line option [4,5,13,14,15,16]. Nevertheless, clinical experience regarding its use in this specific patient population is scarce, and its precise role within treatment algorithms remains undefined.

We hypothesize that milrinone, due to its nitric oxide-independent mechanism and combined inotropic and vasodilatory effects, may serve as an effective alternative or adjunct therapy for PPHN, particularly in resource-limited settings or in neonates unresponsive to iNO. To explore this hypothesis, we present a case series of neonates with PPHN treated with intravenous milrinone in a center without immediate access to iNO. We describe milrinone therapy as a life-saving option in such situations, highlighting the real-world challenges and outcomes across different gestational ages.

Our approach aims to bring a pragmatic perspective to the evolving treatment paradigm, offering insights relevant to clinicians facing similar limitations worldwide. By placing the use of milrinone in a broader context, this work contributes to the ongoing discussion on optimizing care for vulnerable neonates with PPHN in a range of clinical settings. This is accompanied by a review of the available literature focusing on clinical outcomes, safety issues, and potential indications for the use of this drug.

## 2. Materials and Methods

### 2.1. Study Design

#### 2.1.1. Case Series

A retrospective description of three cases of newborns with PPHN hospitalized in the neonatal intensive care unit in 2023–2024, in whom intravenous administration of milrinone was used as an alternative treatment method, was made.

The case series was prepared and reported in accordance with the CARE (CAseREport) checklist to ensure standardized and comprehensive reporting [17]; the completed checklist is provided as Supplementary Table S1. A structured data-collection form aligned with CARE items was used to prospectively abstract patient characteristics, clinical findings, echocardiographic parameters, interventions (timing and dosing), adverse events, and outcomes across prespecified time points (baseline and post-treatment).

#### 2.1.2. Narrative Literature Review

Databases including PubMed, Scopus, and Cochrane Library (until June 2024) were searched using the following terms: “persistent pulmonary hypertension of the newborn”, “PPHN”, “milrinone”, “phosphodiesterase 3 inhibitor”, “inhaled nitric oxide”, “neonate”, “management”, “treatment”. Inclusion criteria comprised English-language publications (2010–2024), including systematic reviews, meta-analyses, randomized clinical trials, case reports, and guidelines from international societies (AHA, ATS, European Society of Cardiology—ESC). Priority was given to studies addressing the efficacy of PPHN treatment. This was a narrative (non-systematic) review; no quantitative synthesis was attempted. Title/abstract screening and full-text selection were performed with a focus on clinical outcomes, safety, dosing, and applicability in resource-limited settings. Reference lists of key articles were hand-searched to identify additional relevant reports.

### 2.2. PPHN Definition

The diagnosis of PPHN was established according to international guidelines [1,2] based on echocardiographic evidence of pulmonary hypertension (e.g., interventricular septal flattening/shift, right ventricular enlargement, tricuspid regurgitation jet velocity—TRV), right-to-left or bidirectional shunting across the ductus arteriosus (DA) and/or foramen ovale (FO), and persistent hypoxemia despite optimized ventilatory support. All echocardiographic assessments were performed by experienced clinicians using standard neonatal views; key parameters (TRV, shunt direction across DA/FO, qualitative LV function, and presence/grade of AV valve regurgitation) were documented at baseline (pre-milrinone) and after initiation of therapy.

### 2.3. Echocardiography and Respiratory Support

Ventilatory mode and settings (including FiO_2_, PIP, PEEP, respiratory rate, inspiratory time) and mean arterial pressure (MAP) were recorded at baseline and during therapy. Echocardiographic re-evaluation was performed after clinical stabilization or within the first 48–72 h of treatment (whichever occurred earlier).

## 3. Results

**Figure 1:**
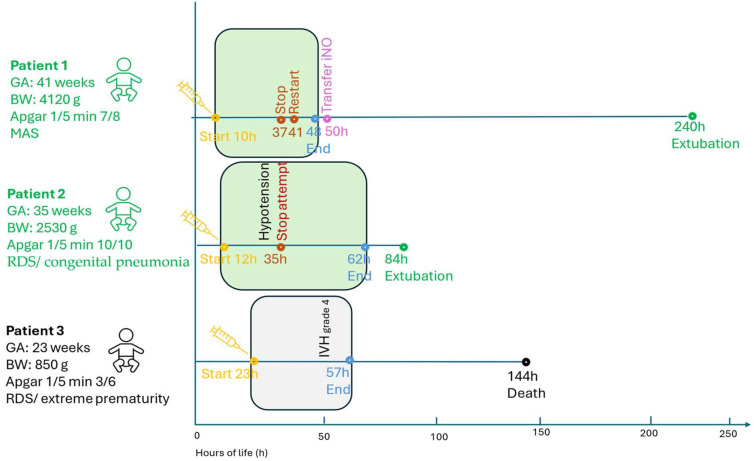
Chronological overview of the clinical course and milrinone therapy in three neonates with PPHN. Each horizontal line represents one patient’s life course (in hours), annotated with timing of milrinone initiation (orange), discontinuation and reinitiation (red), and completion (blue). Green boxes indicate periods of active milrinone infusion. Additional key events include transfer for inhaled nitric oxide (purple, Patient 1), extubation (green circles, Patients 1 and 2), and death following intraventricular hemorrhage (black square, Patient 3). The figure highlights the variability of therapeutic response: transient improvement with relapse in severe-term PPHN (Patient 1), sustained recovery in moderate disease (Patient 2), and limited benefit in extreme prematurity complicated by IVH (Patient 3). Abbreviations: GA—gestational age; BW—birth weight; MAS—meconium aspiration syndrome; RDS—respiratory distress syndrome; IVH—intraventricular hemorrhage; iNO—inhaled nitric oxide; h—hours.

**Figure 2:**
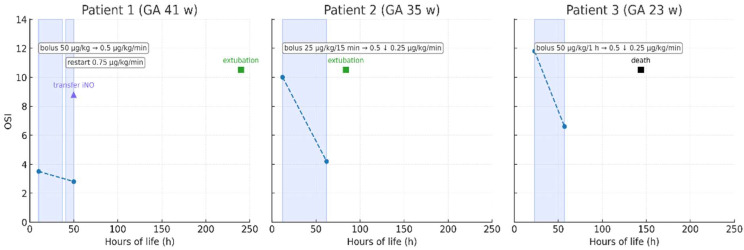
Changes in oxygen saturation index (OSI) following milrinone therapy in three neonates with persistent pulmonary hypertension of the newborn (PPHN). The blue dotted line represents the change in oxygen saturation index (OSI) over time during milrinone infusion.The figure illustrates heterogeneous clinical responses: a transient improvement in severe-term PPHN (Patient 1), a rapid and sustained improvement in moderate disease (Patient 2), and minimal benefit in extreme prematurity (Patient 3). Blue shaded areas correspond to periods of milrinone infusion, with annotated dosing regimens. Triangles indicate transfer for inhaled nitric oxide (iNO; Patient 1), green squares indicate extubation (Patients 1 and 2), and the black square denotes in-hospital death (Patient 3). Abbreviations: OSI—oxygen saturation index; GA—gestational age; iNO—inhaled nitric oxide; h—hours.

Three neonates diagnosed with PPHN between 2023 and 2024 received intravenous milrinone as rescue therapy. The cohort included one term (Patient 1), one late-preterm (Patient 2), and one extremely preterm neonate (Patient 3), representing distinct clinical phenotypes of PPHN. Detailed baseline characteristics and treatment parameters are provided in Supplementary File S1 and Table S1. Echocardiographic findings before and after treatment are summarized in Table 1, while Figure 1 presents the timeline of key therapeutic events. Changes in oxygenation saturation index (OSI) over time are illustrated in Figure 2.

### 3.1. Summary of Patient Characteristics and Clinical Findings

#### 3.1.1. Patient 1 (Term, 41 Weeks, 4120 g)

Developed severe PPHN secondary to meconium aspiration syndrome (MAS) and congenital infection. Despite surfactant, antibiotics, and magnesium sulfate, oxygenation remained poor. Milrinone was initiated at 10 h of life; temporary discontinuation caused deterioration requiring re-initiation at a higher rate. The infant was transferred for iNO therapy at 50 h, later extubated on day 10, and discharged in good condition.

#### 3.1.2. Patient 2 (Late-Preterm, 35 Weeks, 2530 g)

Presented with respiratory distress syndrome (RDS) and congenital pneumonia. Milrinone started at 12 h (0.5 µg/kg/min, reduced to 0.25 µg/kg/min after hypotension). Echocardiographic improvement occurred within 48 h. Extubation was achieved at 84 h, with full recovery and discharge.

#### 3.1.3. Patient 3 (Extremely Preterm, 23 Weeks, 820 g)

Severe RDS and hemodynamic instability due to extreme prematurity. Milrinone initiated at 23 h led to transient echocardiographic improvement but was followed by grade IV intraventricular hemorrhage, coagulopathy, and death on day 6 despite supportive therapy.

### 3.2. Milrinone Administration and Outcomes

Milrinone was initiated between 10 and 23 h of life. Dosing followed neonatal recommendations (bolus 25–50 µg/kg, infusion 0.25–0.75 µg/kg/min) and was titrated according to oxygenation, echocardiographic response, and hemodynamic stability. Primary outcomes included improvement in oxygenation and hemodynamic parameters within 24–72 h; secondary outcomes included successful extubation, transfer to a tertiary center, and survival to discharge (Figure 1).

In the term infant (Patient 1), temporary discontinuation of milrinone led to clinical deterioration, necessitating re-initiation at a higher infusion rate, which resulted in restored oxygenation and hemodynamic improvement. In the late-preterm neonate (Patient 2), transient hypotension required temporary discontinuation and subsequent dose reduction, after which therapy was completed uneventfully. In the extremely preterm infant (Patient 3), severe intraventricular hemorrhage and coagulopathy developed during treatment. These complications were most likely related to extreme prematurity and underlying hemodynamic instability; however, a contributory role of milrinone’s vasodilatory and antiplatelet effects cannot be excluded.

### 3.3. Echocardiography and Respiratory Support

At baseline, echocardiography in all three infants demonstrated clear evidence of PPHN, with tricuspid regurgitant jet velocities (TRV) ranging from 2.7 to 5.1 m/s, right-to-left or bidirectional ductal shunting, and left-to-right flow through the foramen ovale. The term neonate presented with the highest TRV and severe ductal right-to-left flow, while the extremely preterm infant also exhibited depressed left-ventricular systolic function and moderate mitral regurgitation. Mean arterial pressure ranged from 22 to 50 mmHg, indicating variable hemodynamic compromise.

Following milrinone initiation, echocardiographic improvement was observed in all patients within 48–72 h (Table 1). TRV decreased by 20–50%**,** the direction of the ductal shunt became predominantly left-to-right, and the degree of tricuspid regurgitation lessened in both survivors. Left-ventricular systolic performance improved qualitatively, particularly in the term and extremely preterm infants. In the latter, transient recovery of contractility and partial resolution of mitral regurgitation were noted before clinical deterioration related to severe prematurity.

These echocardiographic improvements were accompanied by better systemic oxygenation and lower ventilatory demands. FiO_2_ requirements declined from 0.5–1.0 to 0.3–0.4, and the oxygen saturation index improved accordingly (Figure 2). The term neonate required re-initiation of milrinone after transient worsening upon discontinuation, after which stabilization and recovery ensued. The late-preterm infant improved steadily, allowing extubation on day 4, whereas the extremely preterm neonate experienced temporary echocardiographic response but died on day 6 due to complications unrelated to drug toxicity.

### 3.4. Summary of Findings

All infants demonstrated echocardiographic improvement in pulmonary hypertension markers within 48 h of milrinone therapy, reflected by a decrease in tricuspid regurgitant jet velocity (TRV) and normalization of ductal flow (Table 1). Two neonates achieved sustained clinical improvement and were successfully extubated within 4–10 days. The extremely preterm infant, despite a transient hemodynamic response, succumbed to complications of extreme prematurity and severe intraventricular hemorrhage (IVH).

### 3.5. Literature Review on the Use of Milrinone in PPHN

The 2010 Cochrane Database systematic review revealed a lack of high-quality randomized controlled trials (RCTs) evaluating milrinone in PPHN [18]. Since then, available evidence has remained limited to case series, retrospective analyses, small pilot studies, and a few underpowered RCTs. Table 1 summarizes key studies describing the clinical use of milrinone in neonates with PPHN.

#### 3.5.1. Clinical Outcomes

Most available data originate from case reports [19,20,21], small pilot or observational studies [22,23,24,25], and retrospective analyses [26,27,28,29,30,31,32,33,34]. Across these studies, milrinone administration consistently improved oxygenation indices, right ventricular function, and pulmonary arterial pressures within 24–72 h of initiation.

The only completed RCT, the MINT-1 trial, evaluating adjunct milrinone with iNO, was terminated prematurely because of slow recruitment (*n* = 9; 4 vs. 5 patients) [23]. Another trial investigating dose-dependent effects of milrinone combined with iNO remains ongoing [24]. Despite these encouraging physiological improvements, no study has demonstrated a reduction in mortality, ECMO requirement, or long-term outcomes. Evidence quality remains limited by small sample sizes and methodological heterogeneity.

#### 3.5.2. Safety

The most commonly reported adverse effect is systemic hypotension, particularly after rapid bolus administration or at higher infusion rates [19,21,29]. Hypotension was generally transient and responded to dose adjustment or temporary discontinuation, with no reports of therapy cessation due to severe complications [19,21,25].

Intraventricular hemorrhage (IVH) has been occasionally observed, mainly in critically ill or extremely preterm infants [18], but larger case series and reviews did not demonstrate an increased incidence compared with baseline PPHN risk [27,28]. Nevertheless, given the fragile cerebrovascular autoregulation in very preterm neonates, caution is recommended in this population [29,30].

#### 3.5.3. Dosing

Published studies report relatively consistent dosing regimens, typically a bolus of 25–50 µg/kg over 30–60 min followed by an infusion of 0.25–0.75 µg/kg/min [1,2,14,15,16,19,21].

Because of the prolonged half-life in preterm infants (up to 10 h), rapid bolus administration may increase the risk of hypotension. Dose titration should therefore be gradual, guided by mean arterial pressure and echocardiographic assessment of cardiac output.

Pharmacokinetic variability across gestational ages remains a major limitation to standardizing dosing protocols.

#### 3.5.4. Applicability in Resource-Limited Settings

Several reports emphasize the utility of milrinone in low- and middle-income countries, where iNO or ECMO may be unavailable [14,23,24,25,26,27,30,31,32,33].

Its intravenous formulation, affordability, and NO-independent mechanism make it a pragmatic option for PPHN management under constrained conditions. However, limited monitoring infrastructure increases the risk of unrecognized hypotension or overexposure, underscoring the need for context-adapted protocols and staff training.

Future research in such settings should focus on pragmatic RCTs to determine safe and effective dosing strategies.

## 4. Discussion

This case series and narrative review provide a pragmatic perspective on the use of milrinone for PPHN in a center without immediate access to iNO.

Unlike previous reports from tertiary institutions, our experience spans a wide gestational age range and reflects real-world challenges in resource-limited settings. The observations emphasize that milrinone’s clinical utility is context-dependent and phenotype-specific, highlighting the importance of echocardiographic guidance and individualized hemodynamic assessment.

### 4.1. Phenotype-Specific Responses and Mechanistic Insights

Our findings support the growing recognition that PPHN is not a uniform entity but a spectrum of pathophysiological states. In moderate disease with preserved myocardial function, milrinone produced sustained improvement in oxygenation and right ventricular performance—consistent with prior reports showing rapid hemodynamic stabilization after therapy initiation [19,20,21,23].

In severe-term PPHN, the effect was transient, likely limited by fixed pulmonary vascular remodeling and the absence of synergistic iNO.

In the extremely preterm infant, transient echocardiographic improvement was followed by deterioration and cerebral complications, reflecting the interplay between vascular immaturity, fragile autoregulation, and systemic vasodilation.

These patterns suggest that milrinone responsiveness may vary according to gestational maturity, myocardial involvement, and underlying pulmonary pathology. This aligns with the emerging concept of phenotype-based management in PPHN [34], in which therapies are selected according to dominant mechanisms—maladaptation, maldevelopment, or maladaptive vasoconstriction—rather than a one-size-fits-all approach. Routine use of bedside echocardiography to assess ventricular function, shunt direction, and pulmonary pressures is essential for tailoring therapy and optimizing outcomes. These observations illustrate the challenge of balancing pulmonary vasodilation with systemic perfusion.

### 4.2. Safety Profile and Limitations in Preterm Neonates

Transient systemic hypotension was observed in all infants, consistent with previous reports describing vasodilatory effects of milrinone in neonatal PPHN. No arrhythmias or acute cardiac events occurred. However, in the extremely preterm infant, severe intraventricular hemorrhage and coagulopathy developed, underscoring the need for careful hemodynamic and neurological monitoring during therapy.

#### 4.2.1. Mechanisms and Clinical Context of Systemic Hypotension

Systemic hypotension occurred in all three infants during milrinone therapy, though its pathophysiological background and clinical impact varied with gestational age and disease severity.

In the term neonate (Case 1), hypotension developed after therapy initiation and required vasoactive support, likely reflecting the combined effects of systemic vasodilation induced by milrinone and underlying myocardial dysfunction secondary to hypoxemia and acidosis.

In the late-preterm infant (Case 2), the drop in blood pressure appeared directly related to milrinone administration and resolved promptly after dose reduction, suggesting a primarily dose-dependent pharmacodynamic mechanism.

In the extremely preterm neonate (Case 3), persistent hypotension despite vasoactive support was most likely driven by profound cardiovascular immaturity and systemic inflammation, with milrinone acting as an aggravating rather than primary factor.

These observations highlight the dual origin of systemic hypotension in PPHN—partly inherent to the disease itself and partly related to PDE3 inhibition.

The balance between pulmonary vasodilation and systemic perfusion is strongly influenced by gestational maturity, baseline myocardial function, and adaptive capacity. Therefore, cautious dose titration, avoidance of bolus loading, and continuous blood pressure and echocardiographic monitoring are essential to optimize hemodynamic stability, particularly in preterm infants with limited cardiovascular reserve.

#### 4.2.2. Coagulopathy and Intraventricular Hemorrhage in Extreme Prematurity

In the extremely preterm infant (Case 3), bilateral grade IV intraventricular hemorrhage (IVH) and coagulopathy developed during therapy.

While extreme prematurity, hypoxia, and systemic inflammation were the most likely precipitating factors, the potential contribution of milrinone cannot be excluded.

Its vasodilatory and antiplatelet effects, mediated by increased intracellular cAMP and inhibition of platelet aggregation, may have exacerbated cerebral vulnerability in the context of immature vascular autoregulation and fragile germinal matrix vessels.

Although experimental data suggest that PDE3 inhibition produces only mild antiplatelet effects in term neonates [19,29], this impact may become clinically significant in extremely preterm infants, where systemic vasodilation and baseline coagulopathy coexist.

This case emphasizes the need for individualized risk–benefit evaluation before initiating milrinone in very low birth weight neonates. Avoidance of rapid bolus administration, gradual dose escalation, and close monitoring of coagulation parameters and cerebral perfusion are recommended to minimize bleeding risk in this vulnerable population.

### 4.3. Applicability in iNO-Limited Settings

In many neonatal units, particularly in low- and middle-income countries, access to iNO and ECMO remains restricted. Our experience demonstrates that milrinone may serve as a feasible interim therapy to stabilize pulmonary and systemic hemodynamics. Its use requires continuous hemodynamic and echocardiographic monitoring, skilled interpretation of ventricular function, and readiness to provide vasoactive support in case of systemic hypotension. The variability in responses observed in our cases demonstrates that milrinone’s role depends on gestational maturity, cardiac performance, and disease phenotype. Incorporating this approach into local protocols could help optimize management of PPHN where standard therapies are inaccessible, provided that safety monitoring and individualized dosing are strictly maintained.

### 4.4. Study Limitations and Future Directions

This study is limited by its small sample size, retrospective nature, and lack of a control group, precluding firm conclusions about efficacy or causality. Quantitative echocardiographic parameters were incompletely available, and long-term outcomes were not assessed. Nevertheless, the findings highlight key clinical signals warranting further investigation.

Future prospective, multicenter studies should focus on the following: defining dose–response relationships across gestational ages, evaluating safety and neurodevelopmental outcomes, and assessing milrinone’s cost-effectiveness and practicality in resource-limited settings. Integration of phenotype-guided algorithms with real-time echocardiography could enable precision-based therapy in neonatal PPHN.

## 5. Conclusions

Milrinone improved oxygenation and cardiac performance in term and late-preterm infants with PPHN, serving as an effective bridge therapy when iNO was unavailable.

Systemic hypotension occurred in all cases, reflecting both disease severity and drug-related vasodilation, and requires vigilant monitoring.

In extremely preterm neonates, the risk of IVH and coagulopathy warrants cautious, individualized use.

These findings support a phenotype-specific, echocardiography-guided approach and underline milrinone’s potential as a pragmatic therapeutic option in resource-limited neonatal care.

Further prospective studies are needed to define safe dosing strategies across gestational ages.

## 6. Clinical Implications

Early consideration of milrinone may provide a therapeutic window in centers without immediate access to iNO, potentially stabilizing neonates until definitive treatment options become available.

Integration of phenotype-specific assessment into clinical practice could help identify subgroups of infants most likely to benefit from milrinone treatment, thereby optimizing outcomes while minimizing risks.

## Figures and Tables

**Table 1:** Echocardiography parameters before and after milrinone administration.

	Patient 1		Patient 2		Patient 3	
	Before	After	Before	After	Before	After
TRV (m/s)	5.1	2.6	2.7	2.5	3.0	2.25
DA shunt	R→L	L→R	Bidirectional	L→R	R→L	Bidirectional
FO shunt	R→L	—	L→R	L→R	—	—
LV systolic function (qual.)	not reported	not reported	not reported	not reported	Depressed	Improved
TR (grade)	Mild	Trace	Mild	Trace	not reported	not reported
MR (grade)	not reported	not reported	not reported	not reported	Moderate	Mild

Before therapy, all infants presented with elevated tricuspid regurgitant jet velocity (TRV 2.7–5.1 m/s) and right-to-left or bidirectional ductal flow. Within 48 h of milrinone administration, TRV decreased by approximately 20–50%, and shunt direction normalized (predominantly left-to-right), indicating improved pulmonary hemodynamics. Abbreviations: TRV—tricuspid regurgitant jet velocity; DA—ductus arteriosus; FO—foramen ovale; LV—left ventricle; TR—tricuspid regurgitation; MR—mitral regurgitation; R→L—right-to-left; L→R—left-to-right; qual.—qualitative.

## Data Availability

The original contributions presented in this study are included in the article/Supplementary Material. Further inquiries can be directed to the corresponding author(s).

## Supplementary Materials

The following supporting information can be downloaded at: https://www.mdpi.com/article/10.3390/pediatric17060116/s1, Table S1:Clinical characteristics and treatment course of neonates with PPHN treated with milrinone. Table S2: Summary of published studies and reviews on the use of milrinone in PPHN. File S1: Detailed case descriptions.
