# Supplementary material for: Can Milrinone Be a Therapeutic Alternative in Persistent Pulmonary Hypertension of the Newborn? A Case Series and Narrative Review
Source: Pediatr Rep. 2025 Nov 3;17(6):116. doi: 10.3390/pediatric17060116 (PMC12641953; doi:10.3390/pediatric17060116)
Supplement: Supplementary file 1 [file pediatrrep-17-00116-s001.zip › Supplementary File S1.pdf]

pediatric reports

MDPI

Supplementary File S1. Detailed case descriptions of three neonates with persistent pulmonary hypertension of the newborn (PPHN) treated with intravenous milrinone. Each case includes perinatal background, clinical course, echocardiographic findings, therapeutic interventions, and short-term outcomes.

Patient 1

- Pregnancy and Delivery

A term male neonate (41 weeks of gestation, birth weight 4120 g) was delivered vaginally from the mother's fifth pregnancy (third delivery), complicated by maternal anemia and hypothyroidism. Apgar scores were 8 and 9 at 1 and 5 minutes.

- Postnatal Condition and Initial Management

The infant was presented with severe respiratory failure from birth and was diagnosed with meconium aspiration syndrome (MAS) and congenital infection. Mechanical ventilation was commenced at 4 hours of life, with oxygen saturation maintained at 80–85% despite FiO_2 of 0.5. Initial therapy included surfactant (Curosurf), antibiotics (ampicillin, gentamicin), and magnesium sulfate infusion (20%, for 4–12 hours) without clinical improvement.

- Diagnosis of PPHN

Echocardiography confirmed PPHN with left-to-right flow through the foramen ovale, right-to-left ductal shunting, and mild tricuspid regurgitation.

- Milrinone Therapy and Clinical Course

Milrinone was initiated at 10 hours of life (50 μg/kg bolus, then continuous infusion at 0.5 μg/kg/min), resulting in rapid improvement in oxygenation and ventilation. After 27 hours, the drug was discontinued, but deterioration necessitated reintroduction at 0.75 μg/kg/min. Adjunct therapies included magnesium sulfate and vasopressors.

At 50 hours of life, the infant was transferred to a tertiary center with access to inhaled nitric oxide (iNO). Management included continued mechanical ventilation, iNO, milrinone (up to 46 hours), magnesium sulfate, vasopressors, and targeted antibiotics against methicillin-resistant coagulase-negative staphylococci (MRCNS). Clinical improvement allowed extubation on day 10 and cessation of oxygen therapy on day 12.

Patient 2

- Pregnancy and Delivery

A male neonate was delivered vaginally at 35 weeks of gestation (birth weight 2530 g) following a pregnancy complicated by gestational hypertension and premature rupture of membranes (23 hours). Apgar scores were 10 at both 1 and 5 minutes.

- Postnatal Condition and Initial Management

Respiratory failure developed within 10 minutes after birth. The infant was diagnosed with respiratory distress syndrome (RDS, grade I) and congenital pneumonia. Management included surfactant (Curosurf), antibiotics (ampicillin and gentamicin), and magnesium sulfate infusion (20%, administered for 4–12 hours). Mechanical ventilation was provided using SIMV+PSV (PIP 18, PEEP 5, FiO_2 1.0).

- Diagnosis of PPHN

Echocardiography revealed bidirectional ductal flow, left-to-right flow through the foramen ovale, and tricuspid regurgitation. Oxygenation Saturation Index (OSI) was 10.

- Milrinone Therapy and Clinical Course

Milrinone was administered at 12 hours of life (25 μg/kg over 15 minutes, then 0.5 μg/kg/min, later reduced to 0.25 μg/kg/min due to hypotension). Discontinuation at 35

hours was unsuccessful, and therapy continued for a total of 62 hours. Echocardiographic improvement was observed, with resolution of ductal flow abnormalities and regression of tricuspid regurgitation. Respiratory effort decreased, and extubation was successfully performed at 84 hours of life.

Patient 3

- Pregnancy and Delivery

A female neonate was delivered by cesarean section at 23 weeks of gestation (birth weight 820 g) due to placental abruption, from a pregnancy complicated by placenta previa and maternal anemia. Apgar scores were 3 and 6 at 1 and 5 minutes.

- Postnatal Condition and Initial Management

The neonate presented with extreme prematurity, severe respiratory failure, and was diagnosed with RDS grade II. Initial management included mechanical ventilation, surfactant (200 mg/kg initially, then 100 mg/kg), antibiotics (ampicillin, gentamicin), and magnesium sulfate (200 mg/kg starting from 21 hours of life). Hyperglycemia required insulin therapy during days 1–6.

- Diagnosis of PPHN

Echocardiography at 20 hours showed tricuspid regurgitation (velocity 3 m/s), mitral regurgitation, poor cardiac contractility, and a wide ductus arteriosus with right-to-left shunting. OSI was 11.8.

- Milrinone Therapy and Clinical Course

Milrinone therapy began at 23 hours (50 μg/kg over 1 hour, followed by 0.5 μg/kg/min for 2 hours, then reduced to 0.25 μg/kg/min for 32 hours). Vasopressors (dobutamine, dopamine) were used to maintain mean arterial pressure at 20 mmHg. Respiratory parameters improved (oxygen saturation, reduced FiO_2, decreased respiratory effort). Echocardiography showed improved cardiac contractility, reduced tricuspid regurgitation (2.25 m/s), mild mitral regurgitation, and bidirectional ductal flow.

- Complications

Bilateral grade IV intraventricular hemorrhages (PVHI) developed later. The ductus arteriosus remained hemodynamically significant despite treatment with paracetamol and furosemide (days 3–5). Coagulopathy occurred on days 2–3, requiring vitamin K, fresh frozen plasma, and red blood cell transfusion on day 5. The patient progressed to multiorgan failure and died on day 6 of life.
