# Supplementary material for: Can Milrinone Be a Therapeutic Alternative in Persistent Pulmonary Hypertension of the Newborn? A Case Series and Narrative Review
Source: Pediatr Rep. 2025 Nov 3;17(6):116. doi: 10.3390/pediatric17060116 (PMC12641953; doi:10.3390/pediatric17060116)
Supplement: Supplementary file 1 [file pediatrrep-17-00116-s001.zip › Supplementary Table S1.pdf]

pediatric reports

MDPI

Supplementary Table S1. Baseline characteristics, treatment course, and respiratory parameters of three neonates with persistent pulmonary hypertension of the newborn (PPHN) treated with intravenous milrinone. The table includes perinatal and clinical data, ventilatory settings, dosing regimens, and short-term outcomes. Detailed individual case descriptions are provided in Supplementary File S1.

	**Patient 1**	**Patient 2**	**Patient 3**
Gestational age (weeks)	41	35	23
Birthweight (grams)	4120	2530	820
APGAR (1 min/5 min)	8/9	10/10	3/6
The primary cause of PPHN	MAS	RDS/congenital pneumonia	RDS/extreme prematurity
Cranial ultrasound	No abnormalities	No abnormalities	Bilateral grade 4 IVH
Ventilatory parameters before milrinone administration			
Mode	SIMV	SIMV + PSV	SIMV
PIP (cm H_2O)	16	18	19
PEEP (cm H_2O)	5	5	6
Respiratory rate	50/min	50/min	50/min
Inspiratory time (s)	0.3	0.35	0.3
FiO_2	0.5	1.0	1.0
OSI (max)	3.5	10	11.8
MAP (mmHg)	50	43	22
Treatment			
Surfactant (Curosurf) dose	1	1	2
Sedation<br>Sufentanil (max dose) µg/kg/min<br>Phenobarbital mg/kg/day	Yes<br>0.25<br>0	Yes<br>0.5<br>0	Yes<br>0<br>20
Pressor Amines (max dose)<br>Dobutamine µg/kg/min	Yes<br>10	No<br>0	Yes<br>10

Dopamine μg/kg/min	10	0	16
20% $MgSO_4$ (hours of age)	6-10	4-12	21-22
Milrinone - Start (hours of age)<br>Duration (h)<br>Dose: Bolus (μg/kg) steady<br>infusion (μg/kg/min)	10 (41 restart)<br>27 (7 restart)<br>50<br>0.5 (restart 0.75)	12<br>62<br>25<br>0.5-0.25	23<br>32<br>50<br>0.5-0.25
Outcome			
Transfer to a center with iNO	Yes (50 h of age)	No	No
Extubation (day of life)	10	4	Death on day 6

PPHN – persistent pulmonary hypertension of the newborn, MAS – meconium aspiration syndrome, RDS – respiratory distress syndrome, IVH – intraventricular hemorrhage, $MgSO_4$ – magnesium sulfate, μg/kg/min – micrograms per kilogram per minute, h – hours, SIMV – synchronized intermittent mandatory ventilation, PSV – pressure support ventilation, PIP – peak inspiratory pressure, PEEP – positive end-expiratory pressure, OSI—oxygen saturation index, APGAR – appearance, pulse, grimace, activity, respiration (score at 1 and 5 minutes).
