# Supplementary material for: Can Milrinone Be a Therapeutic Alternative in Persistent Pulmonary Hypertension of the Newborn? A Case Series and Narrative Review
Source: Pediatr Rep. 2025 Nov 3;17(6):116. doi: 10.3390/pediatric17060116 (PMC12641953; doi:10.3390/pediatric17060116)
Supplement: Supplementary file 1 [file pediatrrep-17-00116-s001.zip › Supplementary_Table_S2_Literature_Review.pdf]

pediatric reports

Table S2. Summary of published studies evaluating the use of milrinone in persistent pulmonary hypertension of the newborn (PPHN). The table includes randomized controlled trials, case series, retrospective analyses, and systematic reviews published between 2010 and 2024. Studies are summarized with regard to population characteristics, treatment protocols, clinical outcomes, and reported safety data.

Author / Year	Study Type	Population	Intervention	Outcomes	Conclusion
Case report, RCT, retrospective analysis					
McNamara et al. 2006 [19]	Case series	Term neonates with PPHN un responsive to iNO (n=9)	Milrinone	Improved OI, no significant systemic hypotension	Supportive as rescue therapy
Bassler et al. 2006 [20]	Case series	Neonates with severe PPHN (n=4)	Milrinone	Improved hemodynamics; 3/4 IVH (2 severe cases)	Cautious use advised
McNamara et al. 2013 [21]	Case series	Term neonates with PPHN unresponsive to iNO (n=11)	Milrinone	Improved OI; well tolerated	Supports safety and efficacy
Choobdar et al. 2023 [22]	RCT	Neonates with PPHN (n=31)	Infused vs inhaled Milrinone	Comparable efficacy and safety in both groups	Both routes viable (full recovery: 75% vs 93,3%)
El-Khuffash et al., 2023 [23]	RCT (MINT-1, terminated early)	Neonates with PPHN (n=9)	iNO +Milrinone vs iNO +placebo	No difference, prematurely terminated	Inconclusive
Kirpalani et al. 2023 [24]	RCT -ongoing	Neonates with PPHN	iNO + Milrinone (different doses)	Pharmacokinetic Study of Milrinone	Results not available yet
Dillard et al. 2022 [25]	Retrospective analysis after 5 years	Term neonates with PPHN unresponsive to iNO (n=99)	iNO+ Milrinone vs iNO alone	To identify distinguishing characteristics of neonates with PPHN	Safe and potentially effective as adjuvant therapy to iNO
El-Ghandour et al. 2020 [26]	RCT	Neonates with PPHN	Sildenafil Milrinone vs Sildenafil alone	Faster normalization of pressures, improved gas exchange	Supports combination over monotherapy
Imam et al. 2024 [27]	RCT	Neonates with PPHN >34 weeks (n=40)	Milrinone vs sildenafil	improvements in systolic pulmonary artery pressure and	Milrinone is superior to sildenafil in

				OSI, duration of hospitalization and mechanical ventilation.	improving OSI without lowering blood pressure parameters.
Review					
Bassers et al. 2010 [18]	Cochrane systematic review	Neonates with PPHN	Milrinone	Lack of high-quality RCTs	No firm conclusions
Lakshminrusimha et al. 2016 [14]	Narrative review	Neonates with PPHN	Literature review including milrinone	Summarizes available evidence on milrinone; emphasizes the need for larger trials; no clear survival benefit established	Highlights potential clinical benefits (improve pulmonary vasodilation and cardiac output in iNO-unresponsive PPHN
Qasim et al. 2020 [28]	Narrative review	Neonates with PPHN	Milrinone	Summary of existing literature	Highlights the potential role
Agrawal et al, 2024 [29]	Narrative review	Neonates with PPHN	Milrinone	Potential oxygenation benefit; insufficient survival data	Further trials required
Darren et al. 2024 [30]	Expert review	Preterm neonates with PPHN	Multiple therapies incl. Milrinone	Notes pharmacokinetic variability, potential benefits	Supports used with monitoring in selected patients
Galis et al. 2024 [31]	Scoping review	Neonates with PPHN	Milrinone as mono- or combination therapy	Milrinone as a potential drug in PPHN, both as an adjuvant to iNO as well as a monotherapy	Need for RCTs (especially in low- and middle-income countries)
Matsushita et al.2024 [32]	Systemic review and Meta-analysis	Neonates and children with heart failure and PPHN	Milrinone	Hemodynamic benefit in pediatrics (impact on cardiac, an inotrope and lusitrope function)	potential clinical benefits by improving cardiac function, likely driven by its systemic vasodilatory

					effects. Further trials required
Fei et al. 2024 [33]	Systemic review and Network Meta-analysis	Neonates with PPHN	Multiple therapies incl. Milrinone	evaluate the efficacy and associated mortality of different PPHN therapies	iNO plus sildenafil - is the most effective. If iNO is not available, sildenafil plus milrinone is preferred.
PPHN – persistent pulmonary hypertension of the newborn, iNO – inhaled nitric oxide, RCT – randomized controlled trial, IVH – intraventricular hemorrhage, OI – oxygenation index, OSI - oxygen saturation index					
